# Supplementary material for: A minimal model for microbial biodiversity can reproduce experimentally observed ecological patterns
Source: Sci Rep. 2020 Feb 24;10:3308. doi: 10.1038/s41598-020-60130-2 (PMC7039880; doi:10.1038/s41598-020-60130-2)
Supplement: Supplementary file 1 — Supplementary Information. [file 41598_2020_60130_MOESM1_ESM.pdf]

Supplementary Information

A minimal model for microbial biodiversity can reproduce experimentally observed ecological patterns

Robert Marsland III, Wenping Cui, Pankaj Mehta

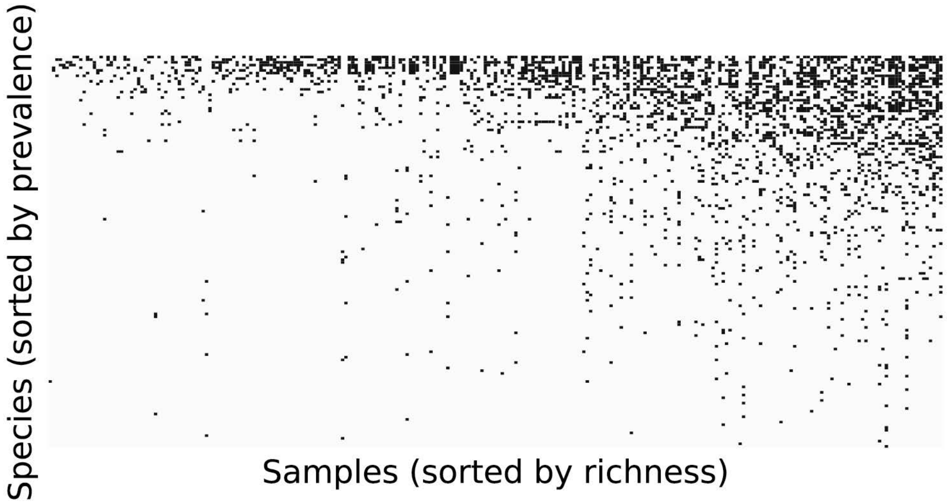

Supplementary Figure S1. Presence (black) and absence (white) of each species in simulations where both the environmental harshness m_env and the initial number of species varied from sample to sample (with sampling of both quantities following the same protocols as in Figure 2 of the main text).

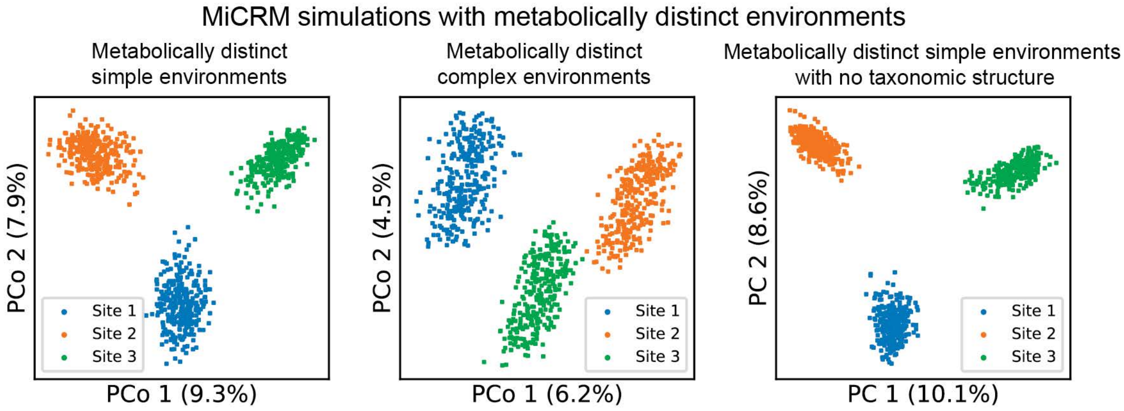

Supplementary Figure S2. PCoA of species-level compositions for the three simulated scenarios in which different body sites were supplied with resources from different classes. See Methods for details.

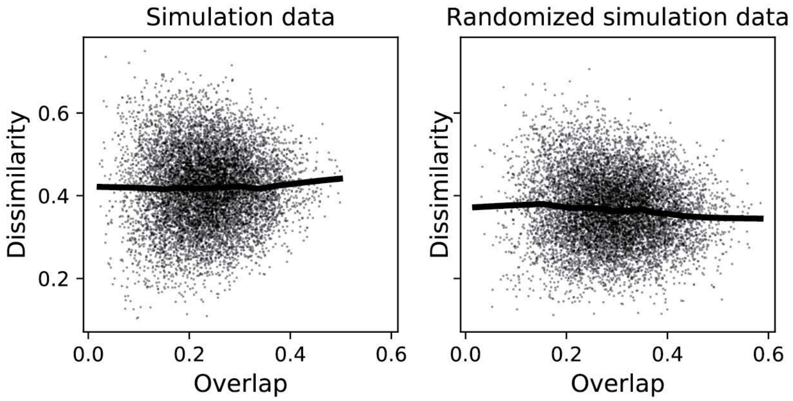

Supplementary Figure S3. Dissimilarity vs. overlap for pairs of samples taken from distinct body sites in the simulated HMP data.
